# Immune System Sex Differences May Bridge the Gap Between Sex and Gender in Fibromyalgia

**DOI:** 10.3389/fnins.2019.01414

**Published:** 2020-01-17

**Authors:** Irene Meester, Gerardo Francisco Rivera-Silva, Francisco González-Salazar

**Affiliations:** ^1^Laboratory of Tissue Engineering and Regenerative Medicine, Basic Sciences Department, University of Monterrey, San Pedro Garza García, Mexico; ^2^Laboratory of Cellular Physiology, Northeast Center of Research, Mexican Institute of Social Security, Monterrey, Mexico

**Keywords:** autoimmune disease, central nervous system sensitization, fibromyalgia, pathophysiology, sex differences, widespread chronic pain

## Abstract

The fibromyalgia syndrome (FMS) is characterized by chronic widespread pain, sleep disturbances, fatigue, and cognitive alterations. A limited efficacy of targeted treatment and a high FMS prevalence (2–5% of the adult population) sums up to high morbidity. Although, altered nociception has been explained with the central sensitization hypothesis, which may occur after neuropathy, its molecular mechanism is not understood. The marked female predominance among FMS patients is often attributed to a psychosocial predisposition of the female gender, but here we will focus on sex differences in neurobiological processes, specifically those of the immune system, as various immunological biomarkers are altered in FMS. The activation of innate immune sensors is compatible with a neuropathy or virus-induced autoimmune diseases. Considering sex differences in the immune system and the clustering of FMS with autoimmune diseases, we hypothesize that the female predominance in FMS is due to a neuropathy-induced autoimmune pathophysiology. We invite the scientific community to verify the autoimmune hypothesis for FMS.

## Introduction

As long as the pathophysiology of the FMS is not elucidated, the diagnosis (Wolfe et al., 2016; Arnold et al., 2019) and the treatment (Macfarlane et al., 2017) will remain inadequate. Many consider FMS to be psychosomatic (Lami et al., 2018) and there are still physicians who do not recognize the disorder. Although the name indicates a fibromuscular affection and the syndrome is classified as a rheumatic disorder, FMS is treated as a neurological problem, in accordance with the currently most accepted hypothesis: central sensitization (Staud et al., 2009). The history of FMS not only reveals the confusion (Inanici and Yunus, 2004) but also the importance of (1) inflammation, (2) a neuropathic type of pain, (3) referred pain after irritation or damage of the paraspinal ligaments, (4) increased substance P levels in cerebrospinal fluid (CSF), and (5) an etiology of trauma and/or infection accompanied by mental stress, which are all consistent with neuroinflammation. Female predominance and clustering with autoimmune diseases were recognized in the historical review (Inanici and Yunus, 2004), but suggestions of autoimmunity markers were omitted (Jacobsen et al., 1990; Klein et al., 1992), despite being actual at the time of the review. Still, it seems that autoimmune susceptibility accompanies FMS. We propose that FMS is a neuropathy-induced autoimmune disease directed to nervous tissue. As autoimmunity is sex biased (Beeson, 1994), the autoimmune hypothesis may explain the female prevalence observed in FMS.

The focus of the paper is to present the biological data from which this hypothesis emerges, followed by how it may explain central sensitization and the sleep alteration that characterize FMS. Next, we describe the mechanisms of immunological self-tolerance and how it can be breached, as well as the well-known sex differences in the immune system, which explains why women are more susceptible to develop certain autoimmunity disorders. We reflect on the complexities of proving the hypothesis and offer suggestions to verify the hypothesis.

## Fibromyalgia: Introducing the Autoimmune Hypothesis

Fibromyalgia syndrome is characterized by unexplained chronic (>3 months) widespread pain accompanied by moderate to severe sleep problems and/or fatigue (Arnold et al., 2019). Fatigue upon awakening has been associated with altered sleep wave patterns, especially a lack of slow-wave sleep (Roizenblatt et al., 2001). A myriad of additional symptoms tends to accompany the disease, amongst them cognitive difficulties, depression, irritable bowel, irritable bladder, restless legs, dry mouth and eyes, and altered sense perception (Arnold et al., 2019). Primary FMS is not accompanied by another chronic pain disorder, whereas secondary FMS develops as a co-morbidity of another dominant chronic disease, commonly an autoimmune disease (Häuser et al., 2015). FMS prevalence among the adult population ranges from 0.8–5% worldwide, depending on the geographical area, case definition, and assessment method (Johnston et al., 2013; Jackson et al., 2015). FMS occurs in the pediatric population, generally beginning with the onset of puberty (Gedalia et al., 2000), but the highest prevalence is among middle-aged women. The female-to-male ratio ranges from 1:1 to 30:1, but a worldwide average is about 3:1 in both the pediatric (Gedalia et al., 2000) and adult populations (Queiroz, 2013).

Although FMS is classified as a musculoskeletal disease, the currently most accepted hypothesis of pathogenesis, central sensitization (Staud et al., 2009), is neurobiology-based and supported by empirical and impartial evidence (Maestu et al., 2013; Sluka and Clauw, 2016). As the etiology and pathophysiology of FMS remain elusive, FMS treatment is directed to symptom management, which includes inhibition of an overreacting CNS (Macfarlane et al., 2017). In general, 30% of the patients report a 30% improvement because of treatment (Häuser et al., 2015). This modest efficacy suggests that the pharmacological treatment does not target the cause.

We hypothesize that FMS is a neuropathy-induced autoimmunity directed against nervous tissue. The autoimmune hypothesis provides a mechanistic explanation for the central sensitization hypothesis and thus the two hypotheses are compatible. Considering sex differences in the immune system, the autoimmune hypothesis may explain female predominance among FMS patients.

**FIGURE 1:**
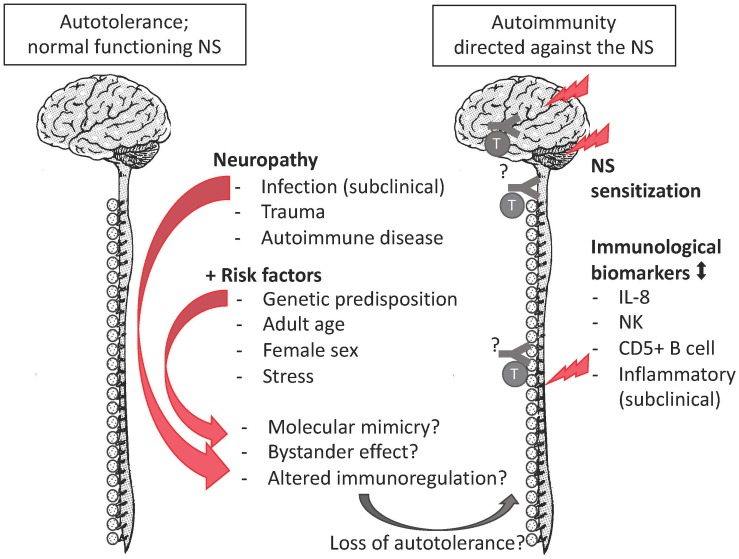
The autoimmune hypothesis for FMS. FMS complies with all mentioned risk factors of autoimmune disease, as well as with research biomarkers of an altered immune response. The missing pieces (indicated by “?”) are the evidence of autoantibodies or autoreactive lymphocytes against nervous tissue.

The autoimmune hypothesis emerged from the following observations. First, the epidemiological profile of FMS is similar to the one of autoimmunity as both peak among middle-aged women (Beeson, 1994). Second, FMS co-occurs with a cluster of autoimmune diseases e.g., sicca syndrome, SLE, rheumatoid arthritis, irritable bowel syndrome, thyroiditis, interstitial cystitis/painful bladder syndrome, and restless legs syndrome co-occur. Autoantibodies ‘specific’ for aforementioned autoimmune diseases tend to be shared rather than unique. When they are detected in FMS, a corresponding autoimmunity is diagnosed and FMS is redefined as secondary FMS (Hervier et al., 2009). Still, secondary FMS reveals autoimmune susceptibility (Buskila and Sarzi-Puttini, 2008; Giacomelli et al., 2013; Haliloglu et al., 2017). Specific antibodies for FMS have been reported (Supplementary Table 1), but they are neither consolidated nor generally accepted (Werle et al., 2001; Giacomelli et al., 2013). Third, there is overlap in the clinical profile of FMS and certain autoimmune diseases, with respect to complex genetic and environmental risk factors. The latter include infections (Smatti et al., 2019) and stress due to traumatic experiences (Sharif et al., 2018). Stress and certain personality characteristics associate positively with autoimmune diseases, FMS, and other chronic diseases in retrospective studies with selected controls (Martin et al., 1996; Lami et al., 2018). But whereas stress and personality are considered precipitating factors or consequences in autoimmune diseases (Mitsonis et al., 2009; Hassett and Clauw, 2010), they are interpreted as the cause in FMS (Lami et al., 2018) despite a lack of convincing evidence demonstrating a causal relation (Häuser and Henningsen, 2014). Similarly, there is no convincing evidence that a certain personality causes pain (Naylor et al., 2017). Fourth, although routinely screened inflammatory biomarkers in FMS samples (such as C-reactive protein levels and erythrocyte sedimentation velocity) tend to be within the normal clinical range, these and other immune markers are significantly different from the ones of healthy controls in a research setting (Kadetoff et al., 2012; Xiao et al., 2013; Pernambuco et al., 2015; Mendieta et al., 2016; Bäckryd et al., 2017; Ciregia et al., 2018). Although data on inflammatory biomarkers are not consisted among studies, they correlate weakly with clinical variables (Ernberg et al., 2018), or can be explained by comorbid conditions, the general impression is that chronic inflammation occurs in FMS (Ernberg et al., 2018). Similarly, routine leukocyte counts are within the normal range, but specific lymphocyte subgroups, not screened in clinical routine studies, are altered in FMS patients. Compared to age-matched healthy controls, female FMS patients had higher proportions of CD57+ natural killer cells (NK) (17.1% *vs*. 11.3%) and CD5+ B cells (6.46% *vs.* 2.5%) (Russell et al., 1999) but lower CD56+ NK (Landis et al., 2004). Case-control observational whole-genome expression studies among women revealed altered expression of immune pathways and markers of tissue destruction (Lukkahatai et al., 2015; Jones et al., 2016). These expression studies did not confirm gene polymorphisms that had been identified in genome association studies (Park and Lee, 2017). The gene association studies often had a selection bias and did not clarify why the genetic susceptibility would only lead to a disorder later in life. In this respect, the *HLA* alleles (Branco et al., 1996; Yunus et al., 1999) form an exception, as their impact depends on an interaction between genetic and environmental factors. *HLA* genes have an essential role in the immune system. Thus, the emerging picture is that a combination of genetic predisposition, a precipitating event (infections, trauma, autoimmune diseases or other reasons of necrosis) (Jiao et al., 2015), and immune dysregulation due to psychological stress (Takahashi et al., 2018) may convert autotolerance or pre-existing occult autoimmunity into overt autoimmunity, but the autoreactive component remains elusive (Figure 1). Importantly, though the precipitation event may be transitory, autoimmunity is a response of the adaptive immune system and is chronic. The sex bias in the immune system may explain the female preponderance of FMS.

## Why Does Central Sensitization Occur?

**FIGURE 2:**
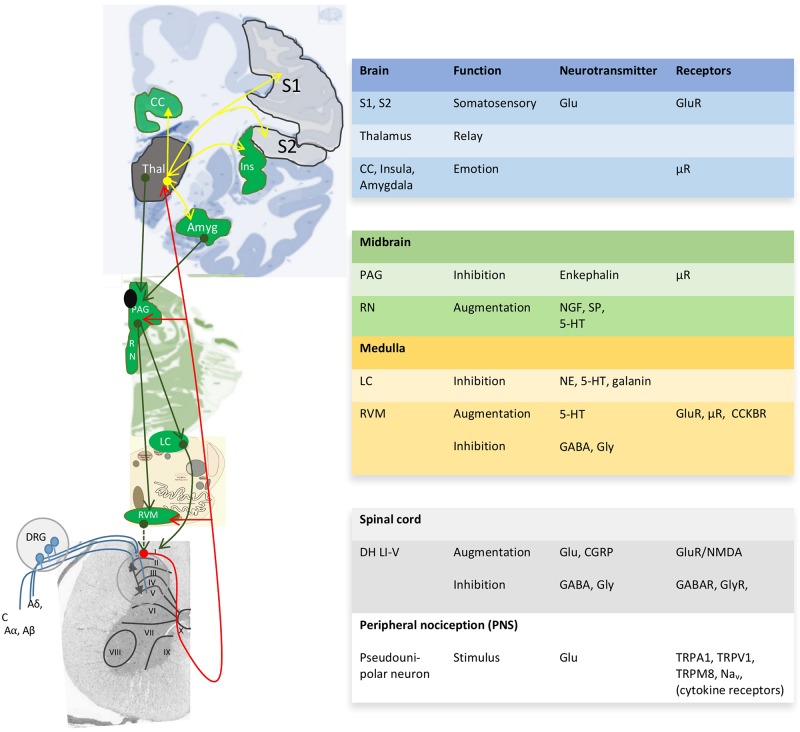
Neuroanatomy and chemistry of the central modulation of pain. Blue projections, incoming signals from 1st order neurons; red projections, ascending projections from 2nd order neurons toward thalamus (Thal) and cortical areas; yellow projections, projections for 3rd order neurons to cortical areas for awareness; green projections, descending projections that modulate the pain pathway. I-X, Reddit layers within the gray matter of the spinal cord; ↔, Integration of modulatory ascending and descending information in the dorsal horn Reddit laminae I-V (DH LI-V). 5-HT, serotonin; Aα, Aβ, Aδ y C, incoming nerves with decreasing levels of myelination; Amyg, amygdala; CC, cingulate cortex; CCKBR, cholecystokinin B receptor; CGRP, calcitonin gene-related peptide; DRG, dorsal root ganglia; Glu, glutamate; Ins, insula; LC, locus ceruleus; Na_*v*_, voltage-gated sodium channels; NE, norepinephrine; NGF, nerve growth factor; PAG, periaqueductal gray; RN, raphe nucleus; RVM, rostroventral medulla; S1, S, somatosensory areas 1 and 2; SP, substance P; TRP, transient receptor potential sensitive to nociceptive stimulus; μR, μ-opioid receptor with high affinity for enkephalins and beta-endorphin. Image based on (Tracey and Mantyh, 2007; Allen Human Brain Atlas, 2010; Ossipov et al., 2010).

Pain perception not only depends on the pain stimulus, but also on the emotional and psychosocial state at a certain moment (Rhudy et al., 2010; Finnern et al., 2018). Both human and animal studies reveal greater pain sensitivity among females than males for most pain modalities (Rhudy et al., 2010; Kisler et al., 2016; Melchior et al., 2016; Aufiero et al., 2017; Kosek et al., 2018). The gender/sex bias in pain perception in various animal species denotes the importance of biological processes and thus sex differences therein. Unfortunately, pain studies aimed at other aspects than a sex or gender bias seldom report outcome variables according to sex or gender and only mention the proportion of males or females among study participants (Supplementary Table 1). As a consequence, although the sex-neutral neurophysiology of the pain pathway is well-described in several reviews (Basbaum et al., 2009; Zeilhofer et al., 2012; Peirs et al., 2015; Kendroud and Hanna, 2019) (Figure 2), little is known about sex differences in pain processing, except for modulation by sex-related hormones (Taleghany et al., 1999; Vincent and Tracey, 2008; Artero-Morales et al., 2018). These biological aspects are at least as important as the psychological aspect (Foo et al., 2017). Though sex differences in functional pain processing in itself are interesting, for FMS the focus is on pathological pain processing, which has been explained with the central sensitization hypothesis.

Central sensitization was originally described as an increased electrophysiological activity in the dorsal horn in both a polyarthritic (Menétrey and Besson, 1982) and a post-injury male rat model (Woolf, 1983). Importantly, in both models a *peripheral* tissue injury triggered off alterations in dorsal horn neurons so that they augment pain signaling to normal input, even from low-threshold Aβ mechanoreceptors (Woolf, 1991). Besides, in animal models, sex differences in pain processing, i.e., at a biological level, could only be detected *after neuropathy* (Sorge et al., 2015) and involved the immune system.

Central sensitization has become the most accepted hypothesis to explain FMS mainly because peripheral sensitization due to autoimmunity is discarded because FMS does not comply with the following criteria of inflammation: (a) the presence of blood inflammatory biomarkers according to common clinical criteria, (b) responsiveness to non-steroid anti-inflammatory cyclooxygenase inhibitors. The former is debatable once research findings on immunological biomarkers (as mentioned in the previous section) are considered. The latter only indicates that FMS is not cyclooxygenase dependent, but discredits neither the involvement of other inflammatory and immune response pathways, nor does it nullify the possibility of neurogenic pain. Failure to comply with inflammatory criteria have not prevented the recognition of Graves’ disease (Baruah and Bhattacharya, 2012) and MS as autoimmune diseases (Luzzio and Dangond, 2018). Furthermore, the need for inflammatory biomarkers does not apply to secondary FMS because the accompanying autoimmune disease will generate not only inflammatory biomarkers but also a continuous nociceptive input. In secondary FMS, the CNS changes appear to improve when nociceptive input is removed (Sluka and Clauw, 2016). The point is that the nociceptive input may also be present in primary FMS, but we are ignorant of it. FMS pain is said to be idiopathic, but the burning, nagging, excruciating pain that is characteristic of FMS (Inanici and Yunus, 2004) is consistent with neuropathy. Histopathological studies have been performed on muscular and connective tissues (Inanici and Yunus, 2004), but not on dorsal root ganglia and central nervous tissue along the pain pathway. History teaches that myasthenia gravis was considered an idiopathic paralysis. Only after having established the involvement of the immune system were pathological immune infiltrates observed in muscle tissue (Hughes, 2005). There are indications that neuropathy is present in FMS patients (Üçeyler et al., 2013; Ramírez et al., 2015; Krumina et al., 2019). FMS pain may reflect a neuropathy of a currently unknown etiology, which is compatible with the central sensitization hypothesis. Latremoliere and Woolf (2009) reviewed neuroplasticity at the molecular and cellular level to explain central sensitization in pain hypersensitivity. They underscore the fundamental contribution of an inflammatory or neuropathic event to initiate the central sensitization.

Rat spinal cord slices exposed to pro-inflammatory cytokines display patch-clamp recordings that are congruent with the central sensitization hypothesis (Kawasaki et al., 2008), suggesting that the immune system plays a role; but, as is the case too often, proper controls were missing. A mouse study revealed the essential role of the immune sensor TLR8 in the maintenance of neuropathic pain. After nerve injury, TLR8 levels increased in the small IB4+ neurons in the dorsal root ganglia and in the ipsilateral dorsal horn. Subsequent intrathecal or intradermal injection of TLR8 agonists (VTX-2337 and miR-21) induced mechanical allodynia, and increased excitability of neurons in the dorsal root ganglia, accompanied by the expression of inflammatory mediators like interleukin 1 beta, interleukin 6, and tumor necrosis factor alpha. These effects were absent or reduced in *TLR8* knock-out mice (Zhang et al., 2018). Aforementioned study did not report on sex differences, but in humans, *TLR8* is an X-linked gene that may escape X-inactivation leading to a dosage difference between men and women (Umiker et al., 2014). TLR8 is especially important because of its pro-autoimmunity potential (Peng et al., 2005). A recent study with BALB/c and C57BL/6 mice suggests that circulating immunoglobulin G (IgG)-type immune complexes may directly mediate hyperalgesia at the level of dorsal root ganglia (Bersellini Farinotti et al., 2019) where macrophages and neurons have receptors for IgG1 and IgG2b (Bersellini Farinotti et al., 2019). If this mechanism were to be confirmed in humans, it would make women more vulnerable, because women are more inclined to humoral adaptive immune responses than men (see Section Sex Differences in the Immune System). Another mouse study revealed sex differences in pain processing. Intrathecal stimulation of the immune sensor of danger TLR4 induced mechanical allodynia in male, but not in female mice. At a cellular level, microglia in the spinal cord proliferated in both sexes after peripheral nerve injury, but only male microglia upregulate the immune sensor of danger, P2RX4 (Mapplebeck et al., 2016). P2RX4 detects nucleotides, mainly ATP, released after CNS stress or injury (Di Virgilio and Sarti, 2018). Activation of P2RX4 receptors leads to release of pro-inflammatory interleukin 1β and brain-derived neurotropic factor, which promote pain hypersensitivity. The inhibition of microglia in the spinal cord reversed allodynia only in male rodents. The female pain process requires more investigation (Sorge et al., 2015). As for humans, sex-biased pain because of knee osteoarthritis could be explained by differences in immune signaling molecules, interleukin 8 and monocyte-chemoattractant protein-1 (Kosek et al., 2018). Interleukin 8 is one of the immunological biomarkers most consistently associated with FMS (Kosek et al., 2015). Thus, sex differences in pathological pain processing mainly involves immune sensors and immune cells. And as we will see in Section “Sex Differences in the Immune System,” the immune system presents sex differences.

Besides, the sex bias of central sensitization remains to be elucidated. We hypothesize that autoimmunity directed to the CNS, either toxic or stimulatory, explains not only central sensitization, but also the female predominance of FMS and the lack of peripheral inflammatory biomarkers.

## Why Is Slow-Wave Sleep Missing?

The neurobiology of sleep and the regulation of the daily sleep-wake cycle have been reviewed with a clinical perspective by España and Scammell (2011). Gender and sex differences in sleep health have been recognized but major gaps continue to exist is areas of sleep regulation, the epidemiology of sleep problems, diagnosis and treatment (Mallampalli and Carter, 2014). Two sexually dimorphic areas, the preoptic area and suprachiasmatic nucleus (Hofman and Swaab, 1989; Hofman et al., 1996) have been associated with sleep problems (España and Scammell, 2011). However, it seems that the sexual dimorphic nucleus of the preoptic area is dedicated to sexual and parental behavior (Rosenblatt et al., 1996) rather than to sleep regulation. More recently, another brain area, the anterior cingulate cortex, has been involved in both primary insomnia (Yan et al., 2018) and FMS (Jensen et al., 2009), but neither study analyzed sex differences. Still, sleep differs between men and women and may contribute to a sex-biased risk for sleep disorders (Mallampalli and Carter, 2014) and, consequently, for FMS.

Polysomnography studies have revealed a variety of sleep disturbances in FMS patients (Choy, 2015). Especially, the phase of deep sleep is reduced and otherwise affected. Instead of the synchronized and therefore high-amplitude slow waves characteristic of the δ rhythm, desynchronized low-amplitude high-frequency waves characteristic of the α rhythm interfere, generating a pattern known as α-δ sleep (Choy, 2015). Deep sleep is considered to be important for memory consolidation and restoration processes. Abnormal deep sleep and other sleep disturbances may contribute to the development of chronic pain (Finan et al., 2013) and FMS (Mork and Nilsen, 2012).

Taking together the difficulty to maintain slow-wave sleep and the peak symptomology upon awakening, it seems as if the regulation of the different sleep phases may be involved. The well-known somnogenic adenosine seems to have a special role in the regulation of the slow-wave sleep phase as revealed by studies with male C57BL/6 mice (Oishi et al., 2017). Extracellular adenosine accumulation activates the adenosine A_1_ receptor which inhibit arousal and induces slow-wave sleep. Another adenosine receptor, A_2__*A*_ receptor, can induce slow-wave sleep, but can be overruled by a motivation stimulus, like hunger or stress (Lazarus, et al. 2019). These receptors are found in the nucleus accumbens. Thus, the nucleus accumbens, already known for being part of the reward circuit, has a role in the control of the slow-wave sleep phase via adenosine receptors. A variety of enzymes and adenosine and nucleotide transporters in both neurons and astroglia are important for extracellular adenosine levels in the micro-environment of the nucleus accumbens. Interestingly, the A_2__*A*_ receptor also regulates naive T cell development in the thymus and its maintenance in the periphery (Cekic et al., 2013). In summary, adenosine, associated metabolites and involved enzymes and transporters may be important in slow-wave sleep and FMS, but further research is necessary.

## Autoimmune Diseases: a Confused Immune System

### Activation and Tolerance in the Immune System

The definition of autoimmune disease is relatively straightforward: a “disease that results when the immune system [..] mistakenly attacks the body’s own tissues” (Wein, 2013). In practice it is more complicated as non-symptomatic healthy persons tend to have autoantibodies (Tan et al., 1997), which are eliminated before doing harm (Nagele et al., 2013). For a disease to be classified as autoimmune there must be *detectable* autoantibodies or autoreactive T-cells in amounts *sufficiently higher than non-symptomatic controls* and they must *explain* the symptoms or *present a strong epidemiological association* with the symptoms. These requirements are fulfilled for all recognized autoimmune diseases, most often because of autoantibodies whether or not in combination with immune infiltrates (Dornmair et al., 2003). In autoimmune diseases that cluster with FMS, such as rheumatoid arthritis, Sjögren’s syndrome, and SLE, specific and diagnostic autoantibodies have been identified. Autoantibodies against cryptic nuclear, cytoplasmic and proteolipid protein antigens are shared in various autoimmune diseases (Suurmond and Diamond, 2015; Fayyaz et al., 2016).

Instead of being due to one specific cause, autoimmune diseases develop when risk factors accumulate. Central to autoimmune diseases is the loss of tolerance to autoantigens. Tolerance of the immune system is the non-activation of the immune response. Upon contact with a substance, particle or pathogen our defense system must decide to attack or to be tolerant. Essential for this decision is recognition, which is different for the three levels of protection of our defense system.

The first line of defense is a biophysicochemical barrier that does not need activation nor recognition. The second level of protection is provided by the innate immune system, a fast-reacting system without memory. Memory is not required as similar cell types or humoral factors share the same receptors for dangers and will attack the same patterns of danger. This in contrast with the third level of protection, provided by T lymphocytes and B lymphocytes. Lymphocytes have unique receptors, so that few will react upon a pathogenic invasion. T lymphocytes are activated by antigen presenting cells of the innate immune system, that provide instructions according to the danger pattern that were encountered. When selected and activated, lymphocytes generate a clone for future memory and effector cells that are either cytotoxic (Tc) or differentiate in a variety of mediators (helper T cells, Th) that potentiate different components of the innate and adaptive immune systems, while effector B lymphocytes (B) become plasma cells (plasma) that produce antibodies. If a tolerance response is erroneously converted into an active immune response, the memory of the adaptive immune response will respond with immune hypersensitivity upon future challenges.

#### Activation and Tolerance in the Innate Immune System

The tolerance mechanism of the innate immune system is passive; i.e., the innate immune system is only activated upon the detection of a limited number of non-self molecular patterns associated with pathogens (PAMPs) or endogenous damage (DAMPs). Hereto, innate immune cells share a limited set of PAMP recognition receptors. Depending on the pattern recognized, an appropriate action is initiated (Hoebe et al., 2004). Everything that is not a PAMP or DAMP is automatically tolerated by the innate immune system. In general, loss of auto-tolerance is not due to breaches of tolerance of the innate immune system.

#### Activation and Tolerance in the Adaptive Immune System

Both recognition/activation and tolerance are more complex in the adaptive immune system (Schwartz, 2012). The adaptive immune system is able to recognize a huge number of structures (known as antigenic determinants or epitopes) because of an enormous variety of specific receptors, T-cell receptors (TCRs) and B-cell receptors, that differ among lymphocytes. As a consequence, when a noxious antigen invades the body, only few specific lymphocytes will react. Clonal proliferation of a triggered lymphocyte generates a specific ‘army’ composed of effector lymphocytes (to eliminate current danger) and memory lymphocytes to provide a faster and stronger immune response for a future challenge with the same antigen.

As the specificity of the TCR and B-cell receptors is generated at random, these receptors may recognize harmless xenoantigens and autoantigens (Klein et al., 2014). To avoid allergies and autoimmune diseases, the adaptive immune system has three control mechanisms: (a) central tolerance, (b) peripheral tolerance, and (c) major histocompatibility complex-restricted activation of T cells, i.e., a T cell can only be activated when its cognate antigen is presented by a major histocompatibility complex molecule.

#### HLA Presents Protein-Associated Epitopes to the Adaptive Immune System

In humans, the major histocompatibility complex is known as HLA. The HLA genes of class I (*A, B*, and *C*) and II (*DP, DQ*, and *DR*) are the most polymorphic genes of the human genome and provide a molecular identity to an individual. All nucleated cells of the human body express HLA-I, although leukocytes express them in larger amounts, while HLA-II is expressed by specialized antigen-presenting cells. HLA-I presents antigens to CD8 + cytotoxic T cells, while HLA-II presents antigens to CD4+ helper T cells (Th) (Hoebe et al., 2004). As antigen presentation to T cells is HLA-restricted, only body-own cells will be protected. The polymorphism of the HLA molecules has an impact on the quality of the immune response. Certain HLA-antigen complexes facilitate the immune response of an individual and others not. Thus, the individuals of a population differ in their protective capacity and their susceptibility to autoimmune diseases. The antigen presentation process is designed for large proteins, preferentially in a particulate presentation. Therefore, electrolytes, sugars, lipids (e.g., sex hormones), nucleic acids, peptides (e.g., neuropeptides), and other small molecules (e.g., neurotransmitters) are passively tolerated. On the other hand, tolerance to protein autoantigens is an active, energy-consuming, and highly controlled process with central and peripheral mechanisms.

#### Mechanisms of Central Tolerance

Central tolerance is established in the primary lymphatic organs, most importantly, in the thymus, where developing T cells or thymocytes reorganize their genome at random to express a unique TCR, either xenoreactive or autoreactive. The thymic epithelial cells function as ‘teachers’ of the thymocytes. Hereto, the thymic epithelial cells express “AIRE),” a transcription factor that facilitates the expression of organ-specific autoantigens in the thymus. The autoantigens are presented in combination with HLA to the thymocytes. To survive, thymocytes should recognize HLA (positive selection) but not recognize autoantigens (negative selection). Simplified, autoreactive thymocytes have two fates (Klein et al., 2014): (1) they die by apoptosis, in case of a strong and long antigen-TCR interaction, and (2) they become regulatory T cells (nTreg), when the antigen-TCR interaction is of intermediate strength and length (Azzam et al., 2001; Ohkura et al., 2013). Thymocytes that do not react with autoantigens or only shortly and with low-affinity are liberated as naïve Th or cytotoxic T cells to protect against danger in the periphery.

As not all autoantigens are expressed in the thymus in sufficient amounts, autoreactive naive T cells may circulate in the periphery, a phenomenon known as ignorance.

#### Mechanisms of Peripheral Tolerance

Peripheral tolerance complements central tolerance by any of the following mechanisms: (1) clonal deletion of autoreactive T cells by apoptosis, (2) the peripheral induction of Treg (iTreg) under the influence of transforming growth factor beta, (3) anergy, i.e., a reversible inactive state of the T cell when the antigen presenting cells do not provide a costimulatory signal (Mueller, 2010), and (4) ignorance, i.e., the amount of auto-antigen is insufficient to induce either tolerance or an immune response. The choice for any of these tolerance options depends on the abundance, strength and duration of the TCR-antigen interactions, but it is conditioned by the absence of a danger signal. As the T cell has no information about the type of danger recognized by its at-random-generated TCR, this information is provided by the antigen presenting cell. Depending on the type of PRR activated the antigen presenting cell provides a costimulatory signal and instructions about the desired type of response (Th1, Th2, or Th17 response) via cytokines (Kaiko et al., 2008). Apart from these major tolerance processes, immune responses are fine-tuned by many stimulatory, inhibitory, and modulatory molecules (e.g., CD5) (Sigal, 2012) and cells, e.g., NKT cells (Dasgupta and Kumar, 2016) within the immune system, as well as neuroendocrine peptides and hormones beyond the immune system (Carniglia et al., 2017). In summary, the adaptive system mainly attacks large protein (>10 kDa) antigens and tolerance to large self-proteins is a complex and highly regulated process.

### Loss of Tolerance and Development of Autoimmune Diseases

Tolerance may be breached for any of the following reasons or a combination of them. First of all, molecular mimicry between an auto-antigen and a pathogenic antigen may confuse the immune system. A well-known example is the Guillain-Barré syndrome due to autoantibodies directed to peripheral nerves because of cross-reaction between nerve autoantigens and certain pathogen antigens, especially *Campylobacter jejuni*, Epstein-Barr virus, cytomegalovirus (Jacobs et al., 1998).

Secondly, bystander autoantigens that are presented in combination with danger signals due to concurrent infections or physical trauma may overcome tolerance, which may play a role in the development of autoimmune diseases (Gestermann et al., 2018). The bystander mechanism may also explain how a ‘founder’ or primary autoimmune disease may lead to secondary autoimmune diseases. Cell lysis due to inflammation of the primary autoimmune disease liberates multiple cryptic autoantigens (mitochondrial antigens, phospholipid antigens, ribonucleoproteins, and other cytoplasmic autoantigens) that provide DAMPs. And the cycle repeats itself: novel autoantigens in combination with danger signals may lead to multiple autoreactive clones of lymphocytes. Cryptic autoantigens liberated during necrosis are shared by different autoimmune diseases (Suurmond and Diamond, 2015). Although the etiology is not proven, latent viral infections associate with MS (Virtanen et al., 2014). A reactivating latent viral infection may cause not only minor damage to nervous tissue but also a breach in tolerance either by molecular mimicry or the bystander effect. Multiple cryptic autoantigens are liberated due to repeated tissue damage, so that polyautoimmunity develops. In MS, oligoclonals are directed against a mixture of autoantigens of cellular debris (Brändle et al., 2016). Initially, MS can be more or less controlled by interferon treatment (Multiple Sclerosis Therapy Consensus Group, 2008), which has antiviral activity, until the polyautoimmune disease has become autosustainable and the relapsing-remitting MS patient turns into a secondary-progressive MS patient. In MS, just as in FMS, inflammatory biomarkers of the blood tend to be within the normal range (Luzzio and Dangond, 2018) as the inflammatory process occurs localized, i.e., behind the blood-brain barrier. Aforementioned process probably plays a role in many autoimmune diseases but have been recognized for only a few. The clinical importance is high because antimicrobial treatment may resolve the initial infection, once the autoimmune disease has developed it may be too late (Mukherjee et al., 2018). Alternatively, it explains why steroid anti-inflammatory treatment worsens (Clark et al., 1985) rather than improves the symptoms.

Thirdly, there is a genetic predisposition for most autoimmune diseases, especially in the HLA genes, probably as a consequence of affinity issues between the HLA molecule and the presented antigen (Bodis et al., 2018). HLA-DR2, DR3, DR4, DQ6, and DQ8 are associated with SLE, sicca syndrome, and rheumatoid arthritis (Mangalam et al., 2013). Furthermore, gene polymorphisms in complement factors may diminish autoantibody clearance (Macedo and Isaac, 2016).

Fourthly, CD5 expression fine-tunes the adaptive immune response in a complex way (Sigal, 2012). CD5 on T or B lymphocytes may either facilitate or inhibit an adaptive immune response depending on avidity issues (Domingues et al., 2016). In general, CD5+ B cells have been related with increased susceptibility for autoimmune disease, while the opposite applies to T cells (Tarakhovsky et al., 1995; Pers et al., 1999; Hawiger et al., 2004).

Fifthly, stress dysregulates the immune system (Sharif et al., 2018). Acute stress enhances catecholamines and circulating leukocytes, which facilitates a stronger immune response. Chronic stress, on the other hand, is immunosuppressive. The stress hormone cortisol diminishes leukocyte numbers and suppresses leukocyte function (Dhabhar, 2008). The stress hormones epinephrine and cortisol induce a rapid leukocyte redistribution. Although the exact mechanisms of this phenomenon remains to be elucidated, clinical and epidemiological data convincingly and consistency reveal an association between chronically stressed people and vulnerability to and resurgence of infections and autoimmune diseases (Reiche et al., 2004).

Sixthly, the immune response is age dependent (Elisia et al., 2017) and age has is a risk factor for loss of central tolerance. In over 60% of autoimmune cases, the onset of symptoms was in the fourth and fifth decade of life, with a median onset at 37.5 years of age (Euesden et al., 2017). A detection bias may play a role in the age effect. Autoimmune disease may not be apparent at the onset, because the progression is slow and their biochemical, physiological or visual detection not obvious. For example, Sjögren syndrome, an autoimmune disease affecting the glands, becomes apparent in older individuals, because destruction of the glands is slow and symptoms of dryness are only experienced when most of the glands are destroyed (Hsu and Mountz, 2003). Importantly, the most essential organ for central tolerance, the thymus, undergoes profound age-associated atrophy (Lynch et al., 2009). Thymic decline is clearly associated with the presence of sex steroids (Heng et al., 2005).

And finally, being female is a risk factor for many autoimmune diseases. A sex bias in the immune system is well-documented and will be described in more detail in the next section.

The unfortunate co-occurrence of aforementioned risk factors elicits an autoimmune disease. It is important to highlight that the initial trigger may be transitory, but the induced autoimmune response is chronic due to the memory of the adaptive immune response. Aforementioned risk factors all apply to FMS (see Section Fibromyalgia: Introducing the Autoimmune Hypothesis and Figure 1).

## Sex Differences in the Immune System

The burden of infectious diseases and the incidence of cancer, allergies, and autoimmune disease differs between men and women. Though this phenomenon can be partially explained by a differential exposure of men and women to environmental risk factors, sex differences in the immune system are evident and well-known. The expression of many immune system-associated proteins and activation of immune cells differs between the sexes as have been reviewed elsewhere (Klein and Flanagan, 2016; Rainville et al., 2018). Well-known is a Th1 bias in male and a Th2 bias in female (Klein and Flanagan, 2016). The latter facilitates the natural passive transfer of immunity from mother to fetus, as immunoglobulins G are able to pass the placenta to protect the fetus. Furthermore, as of adulthood, the number of innate and adaptive leukocytes is higher in females than in age-matched males (Urquhart et al., 2008), except for Treg and innate lymphoid cells, including NK, which are more abundant in males (Klein and Flanagan, 2016). NK have functions beyond cytotoxicity including an important role in the regulation of immune homeostasis and inflammation (Vitale et al., 2005). During aging, the diverse NK population changes gradually; the proportion of immunoregulatory CD56^*hi*^ NK diminishes in favor of highly differentiated cytotoxic CD57^+^ NK. This redistribution may explain functional changes in NK cells with healthy aging (Gayoso et al., 2011). The dysregulation of NK and NKT cells is associated with allergies and autoimmune diseases (Nielsen et al., 2013; Tahrali et al., 2018). Here, we highlight that the cellular immune response tends to predominate in men and the humoral immune response in women. Furthermore, adult men tend to have more immunoregulatory cells than women.

### Differential Effect of Sex Hormones on Leukocyte Behavior

Sex hormones differentially influence the behavior of leukocytes. There is a differential distribution of sex hormones receptors among leukocytes. For example, CD4+ T cells have high levels of the estrogen receptor alpha but low estrogen receptor β levels, whereas the opposite applies for Treg. CD8+ Tc have low expression levels for both types of estrogen receptors, whereas NK express both receptors highly. In mice, high estrogen levels signal via estrogen receptor α and induce antiviral type 1 interferon and NK cells, as well as Treg. Signaling via estrogen receptor β has opposing effect and results in diminished Treg activation. Furthermore, the transmembrane G protein-coupled estrogen receptor, which induces rapid signaling is highly expressed by certain leukocytes (CD4+ Th, Treg, B cells, and macrophages) (Koenig et al., 2017). Estrogen diminishes neutrophil infiltrates and protects against the harmful effects of the innate response (Shimizu et al., 2008; Ritzel et al., 2013). Progesterone and testosterone promote monocyte recruitment and an androgen receptor antagonist reduced monocyte recruitment (Toyoda et al., 2012; Sutti and Tacke, 2018). On the other hand, testosterone treatment reduced immunoglobulin M and immunoglobulin G production and as such diminishes peripheral humoral adaptive immunity (Kanda et al., 1996). The effects of sex hormones are complex because of opposing effects depending on concentration (Hughes, 2012), the variety in metabolic forms that modulate the immune response, and relative concentrations of various sex hormones. Our intention is not to unravel the intricate interactions between the sex hormones and immune system, but to highlight its existence. The area requires more investigation, but it seems that testosterone procures central tolerance. Taken together, testosterone and progesterone downregulate peripheral (humoral) adaptive immunity, but facilitate peripheral innate immunity (Hughes and Clark, 2007; Lai et al., 2012), whereas the opposite applies for estrogen as it is associated with peripheral innate immune suppression, stronger humoral responses, and weaker central tolerance (Kovats, 2015).

### Sex Differences in Immune Tolerance

As mentioned above, the thymus has an essential role in central tolerance. Thymic involution is different between the sexes, especially after the onset of puberty, with a more prominent decline in males than in females, so that the adult female thymus contains more thymocytes and has a higher thymic output than the male thymus (Gui et al., 2012). However, the T cells liberated from the female thymus may have been less well ‘educated’ as the female thymic epitheliocytes express less AIRE and fewer autoantigens (mRNA and protein) than the male ones (Dragin et al., 2016). Low expression levels of AIRE associate with autoimmune disease (Sato et al., 2002; Liu et al., 2014).

The sex hormones, estrogen and dihydrotestosterone (the main active metabolite of testosterone), regulate AIRE expression in opposite directions. At physiological doses, estrogen diminishes AIRE expression, whereas dihydrotestosterone increases AIRE expression (Dragin et al., 2016). Dihydrotestosterone treatment in an experimental animal model of MS, upregulated AIRE and tissue-specific antigen expression in the thymus, improved negative selection of autoreactive T cells, and diminished the severity of autoimmune disease (Zhu et al., 2016). A cross-sectional population-based study revealed that a higher estradiol/testosterone ratio associated significantly with autoimmune thyroid disease among Chinese men (Chen et al., 2017). Interestingly, a clinical phase I/II pilot study among 12 female FMS patients revealed that a 28-day treatment with testosterone gel significantly decreased pain, stiffness, and fatigue (White et al., 2015). Altogether, the efficacy of the thymus depends strongly on age and sex hormones. Male sex hormones seem to compensate for thymic involution with an increased AIRE expression, whereas female sex hormones contribute to diminished tolerance and increased vulnerability to autoimmune diseases.

### Sex Chromosomes and Immune-Associated Genes

The major genetic difference between men and women are the sex chromosomes. Men are XY, whereas women are XX. To enable the pairing of the X and Y chromosomes during male meiosis, small pseudo-autosomal regions are present at the extremes of both the X and Y chromosome. In the pseudo-autosomal regions, the X and Y chromosomes encode the same genes (Mangs and Morris, 2007). For non-pseudoautosomal region genes, males will express the genes of the unique X chromosome, whereas female cells perform at random X inactivation as a dosage compensation mechanism. X inactivation is clonally maintained and generates a functional mosaic organism for X chromosome-encoded genes (Rubtsov et al., 2010). Importantly, X-chromosome inactivation is not an all-or-non-phenomenon; about 10–15% of the X chromosome-encoded genes escape X-inactivation in humans, and a mouse model of accelerated aging revealed increased reactivation with age (Berletch et al., 2011).

Although it has been stated that the X chromosome encodes a disproportionally large number of immune-associated genes (Bianchi et al., 2012), so far no scientifically sound supporting evidence has been published. Still, the increased susceptibility for immune hypersensitivities of men with Klinefelter’s syndrome (XXY) or women with Turner syndrome (X-) reveals the importance of dosage of X-linked immune system-associated genes (Mortensen et al., 2009; Sawalha et al., 2009). An elegant study that used the four genome model (XX*_*Sry*_*+, XY*_*Sry*_*+, XX, XY*_*Sry*_*^–^) in two gonadectomized mouse models for autoimmune disease demonstrated a dosage effect of the X chromosome in SJL mice. Interestingly, the dosage effect was not observed in C57BL/6 mice (Smith-Bouvier et al., 2008). The genetic background of C57BL/6 mice contrast with the one of SJL mice in terms of susceptibility for murine cytomegalovirus and autoimmune disease. In contrast to SJL, C57BL/6 has a bias toward a Th1 response and high NK activity (Sellers et al., 2012; Song and Hwang, 2017). This cellular immune response protects against viral infections.

Two X-linked genes are especially associated with humoral autoimmune disease to cryptic antoantigens. *TLR7* and *TLR8* (both in band Xp22.2) encode endosomal immune sensors that sense microbial and endogenous RNA (Umiker et al., 2014). *TLR7* expression displays a dosage disequilibrium in biallelic B lymphocytes of women and men with Klinefelter’s syndrome. These biallelic B lymphocytes switch more easily to IgG (Souyris et al., 2018), which is consistent with SLE symptom severity in TLR7 + as compared to TLR7-deficient C57BL/6 mice (Desnues et al., 2014). In a 564Igi mouse model of SLE, a dosage difference in TLR8 determined the sex bias in anti-RNA IgG antibodies, which were higher in female than male mice (Umiker et al., 2014). 564Igi mice are especially susceptible to autoimmunity because of diminished somatic hypermutation (McDonald et al., 2017). The release of miR-21 due to neuropathy stimulates TLR8 signaling in the dorsal root ganglia, which leads to hyperexcitability and pain (Zhang et al., 2018). Importantly, TLR8 activation can reverse tolerant Treg into aggressive forms (Peng et al., 2005). Thus, X-linked RNA immune sensors may be activated in neuropathy and favor autoimmunity rather than immune tolerance.

## Autoimmunity to the Nervous System. What Triggers It Off?

The CNS used to be considered an immunoprivileged organ, and therefore little susceptible to autoimmune issues. But this viewpoint has changed over the last two decades upon the detection of autoantibodies against a variety of nervous system autoantigens. As expected, most autoantibodies are directed against large, complex protein autoantigens (Table 1) rather than against small non-protein molecules. Currently, among patients with mental illnesses, the serum prevalence of autoantibodies against nervous tissue antigens is 11–17%, which may be an underestimation (Graus and Santamaría, 2017).

**TABLE 1:** Autoantibodies against nervous tissue autoantigens.

**Autoantibody target**	**Normal function**	**Clinical anomaly**
**Membrane glycolipids**		
GM1, GQ1b	Ganglioside, Schwann cell	Miller-Fisher syndrome, Bickerstaff encephalitis, Guillain-Barré syndrome (1)
MOG, GM, Non-defined	Myelin	Multiple sclerosis, myelin destruction; unmyelinated fibers (2)
**Neurotransmitter receptors**		
Presynaptic VGCC	Voltage-gated calcium channels	Lambert-Eaton syndrome; weak muscles (3)
Postsynaptic nAChR	Nicotinic acetylcholine receptor	Myastenia gravis (4)
AMPAR	Ionotropic glutamate receptor	Limbic encephalitis, seizure, psychosis (5)
GluA3/GluR_3_	Ionotropic AMPAR-type glutamate receptor	Rasmussen encephalitis, unihemispheric brain atrophy (5, 6)
GluN1	Ionotropic NMDAR-type glutamate receptor	Anti-NMDAR encephalitis (psychosis, seizure (5, 6))
mGluR1	Metabotropic glutamate receptor, increase [Ca^2+^]_*intracellular*_	Paraneoplastic cerebellar ataxia (5, 6)
mGluR5	Metabotropic glutamate receptor, release K^+^	Limbic encephalitis, Ophelia syndrome (5, 6)
GABA_*A*_R	Ionotropic GABA receptor; fast-reacting	Encephalitis, seizure (5)
GABA_*B*_R	Metabotropic GABA receptor; slow-reacting	Limbic encephalitis (5)
GlyR	Glycine receptor	Progressive encephalomyelitis with rigidity and myoclonus (PERM); stiff-person syndrome (5)
D_2_R	Pre-synaptic modulatory or post-synaptic dopamine receptor	Limbic encephalitis, seizure, psychosis (5)
**Voltage-gated sodium channels**		
Na(x)	Sodium-sensor and channel	Hypernatremia, neoplasia associated (7)
**Transmembrane proteins or associated protein**		
AQP4	Aquaporin-4, water channel abundant in astrocytes	Neuromyelitis optica (8)
CASPR2	Contactin-associated protein-like 2, transmembrane on axons	- (Limbic) encephalitis, - neuromyotonia, muscle spasms and pain, excessive sweating and disordered sleep - Morvan syndrome - Isaac syndrome, acquired neuromyotonia - fasciobrachial dystonic seizures (3, 5)
VGKC	Voltage-gated potassium channel	
LGI1	Leucine-rich glioma	
DNER	Delta and Notch-like epidermal growth factor-related receptor;	Paraneoplastic cerebellar degeneration (5)
DPPX	Dipeptidyl-peptidase-like protein 6	Encephalitis with diarrhea (5)
DCC	Netrin receptor; involved in axon guidance	Neuromyotonia (5)
IgLON5	Neural cell adhesion molecule	Non-rapid-eye movement and rapid-eye movement parasomnia with abnormal movements and sleep breathing disorder (5, 9)
Neurexin	Presynaptic synapse-facilitating transmembrane protein	Encephalitis (5)
**Cytosolic protein**		
GAD65	Glutamate decarboxylase 65 kD isoform; conversion Glutamate to GABA	Associated with limbic encephalitis, schizophrenia, stiff-man syndrome, diabetes type 1, autoimmune thyroidits, pernicious anemia (3)
GFAP	Glial fibrillary acid protein	Diabetes type 1 (10)
49 kD pituitary cytosolic protein		Autoimmune hypophysitis (11)

*1, Koga et al., 1998; 2, Link et al., 1990; Raddassi et al., 2011; Virtanen et al., 2014; Brändle et al., 2016; 3, Kruse et al., 2015; 4, Noridomi et al., 2017; 5, Fukata et al., 2018; 6, Levite and Ganor, 2008; 7, Hiyama et al., 2010; 8, Jarius and Wildemann, 2013; 9, Sabater et al., 2014; 10, Pang et al., 2017; 11, Caturegli et al., 2005.*

After neuropathy (due to infection or a lesion), intracellular macromolecules that previously were encrypted autoantigens may be exposed and targeted by autoantibodies (Totsch and Sorge, 2017). The latter probably occurs in MS, where oligoclonals target ubiquitous intracellular proteins (Brändle et al., 2016). Oligoclonal bands are interpreted as immunoglobulins that are produced intrathecally, i.e., inside the CNS (Luzzio and Dangond, 2018). Oligoclonal bands occur in 95% of MS patients (Halbgebauer et al., 2016). Other targets of MS autoantibodies are myelin-associated autoantigens (Link et al., 1990) and viral antigens (Virtanen et al., 2014). Although no specific virus is considered to be the causative agent of MS, viruses may be direct or indirect risk factors. The latter via molecular mimicry and/or bystander activation (Virtanen and Jacobsen, 2012) as described in Section “Loss of Tolerance and Development of Autoimmune Diseases.” Various psychiatric diseases are considered to be caused by either an autoimmune process or an infection (Singh and Trevick, 2016; Dubey et al., 2018). We propose that in many cases both occur; the infection would be the initiation event and autoimmunity a consequence. Still they may occur simultaneously, especially when involving opportunistic pathogens. A variety of herpes viruses are opportunistic, pandemic, and neurotropic. Depending on the geographic location, 40–100% of the adult population is infected. A primary infection establishes a lifelong latent infection, which reactivates intermittently without obvious disease symptoms, except for immunocompromised persons. Stress may be a trigger for ‘asymptomatic’ reactivation. Among herpes viruses, cytomegalovirus seems especially apt to alter the immune response into autoimmunity (Andersen and Andersen, 1978; Varani and Landini, 2011; Halenius and Hengel, 2014), while Epstein-Barr virus-transformed lymphocytes tend to produce autoantibodies (Garzelli et al., 1984). In case FMS etiology involves neuropathy by reactivating latent pathogens, the unresponsiveness to corticosteroid treatment is understood. Considering the heterogeneity of FMS patients, other infections or neuropathic events should not be ruled out as possible triggers of autoimmunity.

## The Missing Piece: Evidence of Autoimmune Components Specific for FMS

Autoantibodies against intracellular antigen, nervous and muscle tissue have been reported in FMS patients (Supplementary Table 1), but their role in FMS pathogenesis is controversial. We suggest to screen nervous tissue involved in the pain pathway, both CNS (including the pituitary and pineal glands) and peripheral nervous tissue (dorsal root ganglia) with patient samples (blood and CSF), to complete the missing evidence (Figure 1). A variety of conceptual and technical issues may complicate the detection of autoantibodies. The lack of tissue lesions should not be interpreted as the absence of autoimmunity, as autoantibodies may be stimulatory as in Graves’ disease (Yeung and Habra, 2018). Screening of autoantibodies should not be limited to blood, as autoantibodies or oligoclonals may be limited to CSF when neuropathic symptoms predominate (Luzzio and Dangond). Furthermore, pleocytosis of leukocytes in CSF should be evaluated. Autoantibody screening on certain tissues may yield false negatives when the autoantibodies are directed against other tissues than the ones that are screened. Screening on animal tissues may yield false negatives when the human autoantigens are sufficiently different from the animal forms. Screening on fixed tissues may yield false negatives because the appropriate antigen retrieval method was not applied. Also, the autoimmune response may be cellular rather than humoral. A conceptual or interpretation issue are prodromal autoantibodies; tissue destruction mediated by prodromal autoantibodies remains asymptomatic until the overcapacity of the targeted organ has been lost (Arbuckle et al., 2003; Hayashi et al., 2008; Haller-Kikkatalo et al., 2017). During the prodromal period, autoantibodies run the risk to be interpreted as false positives. Longitudinal follow-up studies of patients with prodromal autoantibodies would be interesting. And finally, because of the heterogeneity among FMS patients, a certain etiology or pathogenesis may be limited to a subgroup of FMS patients (Jacobsen et al., 1990; Purnamawati et al., 2018). The worst scenario would be that the detected pathogenesis is discarded because it does not apply to a sufficiently high proportion of FMS patients. To avoid this situation, stratification or clustering of FMS patients is recommendable. Despite the aforementioned, the challenge is not impossible; the detection of anti-IgLON5 is exemplary (Sabater et al., 2014). We recommend a similar screening technique to verify whether an autoimmune process is involved in the pathogenesis of FMS.

## Conclusion

The clinical profile of FMS displays a strong overlap with certain autoimmune diseases. In fibromyalgia, physical or mental stress may constitute a precipitating factor or a consequence rather than a cause, similar to the situation in autoimmune diseases. Stress may debilitate the immune system and allow for reactivation of a latent (viral) infection, which may cause neuroinflammation or neuropathy and facilitate autoimmune phenomena. However, different from most autoimmune diseases, common clinical serum markers of inflammation are within the normal range in FMS. Still, altered immunological biomarkers, especially CD57 and IL-8 levels, are compatible with a viral infection or autoimmune mechanism. Sex differences in the immune system would explain a sex bias in FMS prevalence. If convincing evidence for an autoimmune process were detected for FMS, diagnostic tests and effective therapies could be developed. Blood and CSF should be screened for autoantibodies and/or autoreactive lymphocytes. Screening for autoantibodies directed to peripheral nervous tissues and CNS should include dorsal root ganglia, the spinal cord, pituitary gland and pineal gland, should be screened as possible targets for autoantibodies and autoreactive lymphocytes.

## Author Contributions

All authors complied with the ICMJE criteria for authorship of contribution in conception or data acquisition, drafting or revising the manuscript, approval of the final document, and agreement with all aspects of the document. The contributions of GR-S were mainly in the neuroscience and pain part, the ones of FG-S in the immune system section, and IM in the FMS and autoimmune aspects, as well as the coordination of the publication aspects.

## Conflict of Interest

The authors declare that the research was conducted in the absence of any commercial or financial relationships that could be construed as a potential conflict of interest.

## Abbreviations

AIRE: autoimmune regulator
CNS: central nervous system
CSF: cerebrospinal fluid
FMS: fibromyalgia syndrome
HLA: human leukocyte antigen
MS: multiple sclerosis
NK: natural killer cell
SLE: systemic lupus erythematosus
TCR: T cell receptor
TLR: Toll-like receptor
Th: helper T lymphocyte
Treg: suppressor T lymphocyte.
