# Supplementary material for: Immune System Sex Differences May Bridge the Gap Between Sex and Gender in Fibromyalgia
Source: Front Neurosci. 2020 Jan 17;13:1414. doi: 10.3389/fnins.2019.01414 (PMC6978848; doi:10.3389/fnins.2019.01414)
Supplement: Supplementary file 1 [file Data_Sheet_1.pdf]

Supplementary Table 1 Attention for sex differences in pain and FM research

Essentials of study design	Species	Total sample size (n) or size/group	% ♀	Separate analysis of sex or gender	Comment	Study conclusion
**Pain perception**						
Self-reported pain after pain stimulus in CLBP patients (1)	H	319	47	Yes		Self-reported pain higher in women.
Pain self-report and association with cytokine levels (2)	H	Aprox. 40/group	43-58	Yes	Sex difference in immune system associates with sex bias in pain perception	Self-reported pain higher in women with immune issues
Pain VAS & Likert after pain stimulus (3)	H	HC: 100	59	Yes		Self-reported pain higher in women.
Contact heat-evoked potential measurement (4)	H	26	50	Yes		Emotion-dependent pain sensitivity higher in women.
Electrophysiological rx after post-emotional pain stimulus (5)	H	120	61	Yes		Emotion alters pain perception but does not explain sex bias
Meta-analysis (6):<br>Pain perception<br>Heart rate,<br>Blood pressure,<br>Electrodermal rx<br>Noxious inhibitory controls, variety	H	2269	50	Yes	Many studies are unreliable (too small; invalid results, etc.)	Insufficient evidence for biopsychological factors as basis for sex bias in pain perception; perhaps social factors.
Noxious injection in gonadectomized mice (7)	A	6/group	50	Yes	Small groups.<br>Sex bias in pain perception in animals directs towards a biological mechanism.	Pain sensitivity higher in females.
Review (8)	H & A	NA	NA	Yes	Reviewed studies are inconsistent in design and results.	Females more sensitive. Underlying mechanism unknown.
Electrophysiology after neuropathy (9a & 9b)	A	?	?	No	First physiological evidence of central sensitization	Central sensitization
Multiple after neuropathy (10)	A	4-6/sex/ condition	50	Yes	Immunology concept explains sex bias in pain perception	Involvement microglia and TLR4 in male but not female pain perception
Multiple after neuropathy (11)	A	3-5/ group	?	No	Immunology based	TLR8 involvement
Sex differences in immune system						
Meta-analysis (12); multispecies	A	NA		Yes	Well-known	Females have a stronger immune response.
Meta-analysis (13) multispecies	A	NA		Yes	Well-known	Sex bias in immunity exists in many

						animals, especially adults.
Review (14)	H			Yes	Well-known	Mechanisms of sex bias in innate immunity.
Review (15)	H			Yes		Sex affects immunity. (X-linked genes, hormones)
Review (16)	H			Yes	Well-known.	Sex bias in autoimmunity.
Fibromyalgia pathophysiology						
Ultrasonography<br>LANSS (17)	H	242<br>FM: 51<br>P: 51<br>PA: 140	 92<br>55<br>50	No	Healthy control missing	1 enthesis abnormality common<br>PA: 92%, P: 90%, FM: 75%<br>FM: LANSS+
In vivo confocal microscopy<br>LANSS (18)	H	34<br>FM: 17<br>HC: 17	100	No	Small n,<br>p = 0.01 or 0.02	Thinner corneal stromal nerves and diminished sub-basal plexus nerve density in FM<br>FM: LANSS+
Pain VAS & ^{1}H-MR spectroscopy (19)	H	48<br>FM: 21<br>HC: 27	 81<br>78	 No	small n,<br>no diagnostic relevance	Difference in relative metabolite amount between FM and HC
Self-reported pain and neuroimaging after pain; (20)<br>FM vs HC	H	FM: 16<br>HC: 13	94	No		FM stronger pain perception
CRP levels;<br>Comparison FM *vs.* non-FM (21)	H	52535<br>FM 1125<br>FM: 51410	 92	No	Non-FM may have other problems that increase CRP	CRP and SR increased in FM
Case-control, pain-related evoked potentials<br>Pathology of unmyelinated nerve fibers only in FM (22)	H	FM: 25<br>Ps: 10<br>HC: 10	82<br>80<br>?	No	Confirmatory experiments included.<br>Insufficient men	Neuropathy-induced pain in FM
Comparison proinflammatory biomarkers in<br>FM *vs*. HC (23)	H	105	91	No		FM: increased CRP and SR, but not IL-6 nor IL-8
Comparison proinflammatory biomarkers in<br>FM *vs*. HC (24)	H	28<br>FM: 15<br>HC: 13	93	No	Small, confirmation required	IL-6, IL-8 higher in FM
AutoAb screening via ELISA, RIA, MHA<br>Indirect IF (sera dilutions: 1:10-1:1000) (25)	H	39<br>FM: 20<br>HC: 19	100	No	Small groups,<br>No confirmatory studies	Anti-muscle autoAb in 40%-55% PFM patients; absent in HC
IF on cryostat sections on rat liver, stomach, and kidney. (26)	H	478<br>FM: 223<br>HC: 255	 90<br>29	 No	Prejudice perceivable in study design.<br>Screened on rat tissue;<br>HC not sex nor age matched.	No autoAb in PFM
AutoAb screening via ELISA and IF (patient sera at 1:500-1:1000)<br>(27)	H	FM: 50<br>CF: 42<br>AID: 62<br>NP: 128<br>HC: 32	82<br>?<br>?<br>?<br>?	No	Well-designed.	Anti-serotonin and anti-ganglioside autoAb in 54%- 73% PFM *vs*. max. 25% in non-FM AID

AutoAb screening via ELISA (28)	H	203 FM<br>64 HC		Si	Confirmation anti-serotonin autoAb, but no diagnostic significance	Anti-serotonin autoAb: 20% FM, 5% non-pain CTR
AutoAb screening via on HeLa extract via immunoblot (29)	H	PFM: 90<br>SFM: 35<br>CF: 114<br>AID: 19<br>Pi: 37<br>HC: 37	86<br>91<br>82<br>95<br>82<br>82	No	Test titer immunoblot unknown;<br>No confirmatory test, nor follow-up.<br>Difficult to interpret.	Anti-68/48 kD autoAb in 16% PFM, 13% CF;<br>Anti-45 kD autoAb in 22-37% SFM, Ps;<br>These autoAb are absent in AID and HC.
AutoAb screening via ELISA (30)	H	 FM: 39<br>SLE: 17<br>HC: 19	92<br>94<br>53	No	Test titer unknown;<br>No significantly different from HC<br>No diagnostic value	Anti-DFS70 autoAb associate with sleep disturbances and pain in FM
Screening for Sjögren-related autoAb at clinical lab(31)	H	FM: 185	93	No	Suggests that autoimmunity is involved in FM pathogenesis	30% FM patients have Sjögren-related autoAb

A, animal study; AID, autoimmune disease; autoAb, autoantibody; CF, chronic fatigue; CLBP, chronic low back pain; CRP, C-reactive peptide, ELISA, enzyme-linked immunosorbent assay; FM, fibromyalgia; H, human study; HC, healthy control; ^{1}H-MR, proton magnetic resonance; IF, immunofluorescence; IHC, immunohistochemistry; LANSS+, neuropathy according to Leeds Assessment of Neuropathic Symptoms and Signs; MHA, microhemagglutination; NP, neuropathy; rx, reaction; P, psoriasis; PA, psoriatic arthritis; PFM, primary FM; Pi, psychiatric issues; RIA, radioactive immunosorbent assay; SFM, secondary FM; SLE, systemic lupus erythematosus; SR, sedimentation rate; VAS, visual analogue scale.

References

1 Meints, S. M., Wang, V., and Edwards, R. R. (2018). Sex and Race Differences in Pain Sensitization among Patients with Chronic Low Back Pain. *J. Pain.* 19, 1461-1470. doi:10.1016/j.jpain.2018.07.001
2 Kosek, E., Finn, A., Ultenius, C, Hugo, A., Svensson, C, and Ahmed, A. S. (2018). Differences in neuroimmune signalling between male and female patients suffering from knee osteoarthritis. *J. Neuroimmunol.* 321, 49-60. doi: 10.1016/j.jneuroim.2018.05.009
3 Aufiero, M., Stankewicz, H., Quazi, S., Jacoby, J., and Stoltzfus, J. (2017). Pain Perception in Latino vs. Caucasian and Male vs. Female Patients: Is There Really a Difference? *West. J. Emerg. Med.* 18, 737-742. doi: 10.5811/westjem.2017.1.32723
4 Kisler, L. B., Granovsky, Y., Sinai, A., Sprecher, E., Shamay-Tsoory, S., and Weissman-Fogel, I. (2016). Sex dimorphism in a mediatory role of the posterior midcingulate cortex in the association between anxiety and pain sensitivity. *Exp. Brain Res*. 234, 3119-3131. doi: 10.1007/s00221-016-4710-9
5 Rhudy, J. L., Bartley, E. J., Williams A. E., McCabe, K. M., Chandler, M. C., Russell, J. L., and Kerr, K. L. (2010). Are there sex differences in affective modulation of spinal nociception and pain? J. Pain. 11, 1429-1441. doi: 10.1016/j.jpain.2010.04.003
6 Racine, M., Tousignant-Laflamme, Y., Kloda, L. A., Dion, D., Dupuis, G., and Choinière, M. (2012). A systematic literature review of 10 years of research on sex/gender and experimental pain perception – part 1: are there really differences between women and men? *Pain.* 153, 602-618. doi: 10.1016/j.pain.2011.11.025
7 Sluka, K. A. and Rasmussen, L. A. (2010). Fatiguing exercise enhances hyperalgesia to muscle inflammation. *Pain.* 148, 188-197. doi: 10.1016/j.pain.2009.07.001
8 Melchior, M., Poisbeau, P., Gaumond, I., and Marchand, S. (2016). Insights into the mechanisms and the emergence of sex-differences in pain. *Neuroscience* 338, 63-80. doi: 10.1016/j.neuroscience.2016.05.007
9a Menétrey, D. and Besson, J. M. (1982). Electrophysiological characteristics of dorsal horn cells in rats with cutaneous inflammation resulting from chronic arthritis. *Pain.* 13, 343-364.
9b Woolf, C. J. (1983). Evidence for a central component of post-injury pain hypersensitivity. *Nature.* 306, 686-688
10 Sorge, R. E., Mapplebeck, J. C. S., Rosen, S., Beggs, S., Taves, S., Alexander J. K., et al. (2015). Different immune cells mediate mechanical pain hypersensitivity in male and female mice. *Nat Neurosci.* 18, 1081-1083. doi: 10.1038/nn.4053
11 Zhang, Z-J., Guo, J-S., Li, S-S., Wu, W-B., Cao D-L., Jiang, B-C., et al. (2018). TLR and its endogeneous ligand miR-21 contribute to neuropathic pain in murine DRG. *J Exp. Med*. 215, 3019-3037. doi: 10.1084/jrem.20180800

12 Foo, Y. Z., Nakagawa, S., Rhodes, G., and Simmons, L. W. (2017). The effects of sex hormones on immune function: a meta-analysis. *Biol. Rev. Camb. Philos. Soc*. 92, 551-571. doi: 10.1111/brv.12243
13 Kelly, C. D., Stoehr, A. M., Nunn, C., Smyth, K. N., and Prokopp, Z. M. (2018). Sexual dimorphism in immunity across animals: a meta-analysis. *Ecol. Lett*. 21, 1885-1894. doi: 10.1111/ele.13164
14 Jaillon, S., Berthenek, K., and Garlanda, C. (2019). Sexual dimorphism in innate immunity. *Clin. Rev. Allergy Immunol*. 56, 308-321. doi: 10.1007/s12016-017-8648-x
15 Pennell, L. M., Galligan, C. L., and Fish, E. N. (2012). Sex affects immunity. J. Autoimmun. 38, J282-291. doi: 10.1016/j.jaut. 2011.11.013
16 Purnamawati, K., Ong, J. A., Deshpande, S., Tan, W. K., Masurkar, N., Low, J. K., et al. (2018). The importance of sex stratification in autoimmune disease biomarker research: a systematic review. *Front. Immunol*. 9, 1208. doi: 10.3389/fimmu.2018.01208
17 Macchioni, P., Salvarani, C., Possemato, N., Gutierrez, M., Grassi, W., Gasparini, S., et al. (2019). Ultrasonographic and clinical assessment of peripheral enthesitis in patients with psoriatic arthritis, psoriasis, and fibromyalgia syndrome: The ULISSE study. *J Rheumatol*. 46, 904-911. doi: 10.3899/jrheum.171411.
18 Ramírez, M., Martínez-Martínez, L.A., Hernández-Quintela, E., Velazco-Casapía, J., Vargas, A., and Martínez-Lavín, M. (2015). Small fiber neuropathy in women with fibromyalgia. An in vivo assessment using corneal confocal bio-microscopy. *Semin. Arthrit., Rheum.* 45, 214-219. doi: 10.1016/j.semarthrit.2015.03.003
19 Petrou, M., Harris, R. E., Foerster, B. R., McLean, S. A., Sen, A., Clauw, D. J., et al. (2008). Proton MR spectroscopy in the evaluation of cerebral metabolism in patients with fibromyalgia: comparison with healthy controls and correlation with symptom severity. *AJNR Am J Neuroradiol*. 29, 913-918. doi: 10.3174/ajnr.A0959
20 Gracely, R. H., Petzke, F., Wolf, J. M., and Clauw, D. J. (2002). Functional magnetic resonance imaging evidence of augmented pain processing in fibromyalgia. *Arthritis Rheum*. 46, 1333-1343. doi: 10.1002/art10225
21 Feinberg, T., Sambamoorth, U., Lilly, C. and Innes, K. K. (2017). Potential mediators between fibromyalia and C-reactive protein: results from a large U.S. community survey. *BMC Musculoskelet Disord.* 18, 294. doi: 10.1186/s12891-017-1641-y
22 Üçeyler, N., Zeller, D., Kahn, A. K., Kewenig, S., Kittel-Schneider, S., Schmid, A., et al. (2013). Small fibre pathology in patients with fibromyalgia syndrome. *Brain*. 136, 1857-1867. doi: 10.1093/brain/awt053
23 Xiao, Y., Haynes, W. L., Michalek, J. E., and Russell, I. J. (2013). Elevated serum high-sensitivity C-reactive protein levels in fibromyalgia syndrome patients correlate with body mass index, interleukin-6, interleukin-8, erythrocyte sedimentation rate. *Rheumatol. Int*. 33, 1259-1264. doi: 10.1007/s00296-012-2538-6
24 Mendieta, D., De la Cruz-Aguilera, D. L., Barrera-Villalpando, M. I., Becerril-Villanueva, E., Arreola, R., Hernández-Ferreira, E., et al. (2016). IL-8 and IL-6 primarily mediate the inflammatory response in fibromyalgia patients. *J. Neuroimmunol*. 290, 22-25. doi: 10.1016/j.jneuroim.2015.11.011
25 Jacobsen, S., Hoyer-Madsen, M., Danneskiold-Samsoe, B., and Wiik, A. (1990). Screeningn for autoantibodies in patients with primary fibromyalgia syndrome and a matched control group. *APMIS*. 98, 655-658
26 Bengtsson, A., Emerudh, J., Vrethem, M., and Skogh, T. (1990). Absence of autoantibodies in primary fibromyalgia. *J Rheumatol.* 17, 1682-1683.
27 Klein, R., Bänsch, M., and Berg, P. A. (1992). Clinical relevance of antibodies against serotonin and gangliosides in patients with primary fibromyalgia syndrome. *Psychoneuroendocrinol*. 17, 593-598.
28 Werle, E., Fischer, H. P., Müller, A., Fiehn, W., and Eich, W. (2001) Antibodies against serotonin have no diagnostic relevance in patients with fibromyalgia syndrome. *J. Rheumatol*. 28, 595-600.
29 Nishikai, M., Tomomatsu, S., Hankins, R. W., Takagi, S., Miyachi, K, Kosaka, S., et al. (2001). Autoantibodies to a 68/48 kDa protein in chronic fatique syndrome and primary fibromyalgia: a possible marker for hipersomnia and cognitive disorders. *Rheumatol.* 40, 806-810. doi: 10.1093/rheumatology/40.7.806
30 Jeong, J., Kim, D. H., Park, G., Park, S., and Kim, H. S. (2017). Clinical significance of anti-dense fine speckled 70 antibody in patients with fibromyalgia. *Korean J, Intern. Med*. 34, 426-433 doi: 10.3904/kjim.2016.276
31 Applbaum, E. and Lichtbroun, A. (2019). Novel Sjögren´s autoantibodies found in fibromyalgia patients with Sicca and/or Xerostomia. *Autoimmun. Rev*. 18, 199-202. doi: 10.1016/j.autrev.2018.09.004
